# Systematic identification and characterization of long intergenic non-coding RNAs in fetal porcine skeletal muscle development

**DOI:** 10.1038/srep08957

**Published:** 2015-03-10

**Authors:** Weimin Zhao, Yulian Mu, Lei Ma, Chen Wang, Zhonglin Tang, Shulin Yang, Rong Zhou, Xiaoju Hu, Meng-Hua Li, Kui Li

**Affiliations:** 1 The State Key Laboratory for Animal Nutrition, Institute of Animal Science, Chinese Academy of Agricultural Sciences, Beijing 100193, China; 2 CAS Key Laboratory of Animal Ecology and Conservation Biology, Institute of Zoology, Chinese Academy of Sciences (CAS), Beijing 100101, China; 3 University of Chinese Academy of Sciences, Beijing 100049, China

## Abstract

Long intergenic non-coding RNAs (lincRNAs) play important roles in many cellular processes. Here, we present the first systematic identification and characterization of lincRNAs in fetal porcine skeletal muscle. We obtained a total of 55.02 million 90-bp paired-end reads and assembled 54,550 transcripts using cufflinks. We developed a pipeline to identify 570 multi-exon lincRNAs by integrating a set of previous approaches. These putative porcine lincRNAs share many characteristics with mammalian lincRNAs, such as a relatively short length, small number of exons and low level of sequence conservation. We found that the porcine lincRNAs were preferentially located near genes mediating transcriptional regulation rather than those with developmental functions. We further experimentally analyzed the features of a conserved mouse lincRNA gene and found that isoforms 1 and 4 of this lincRNA were enriched in the cell nucleus and were associated with polycomb repressive complex 2 (PRC2). Our results provide a catalog of fetal porcine lincRNAs for further experimental investigation of the functions of these genes in the skeletal muscle developmental process.

In mammals, a large proportion of the genome is composed of intergenic regions, yet little was known about the transcription of these regions at the time of completion of the human genome^1^. Several studies in the past decade, however, have revealed that most of these regions may represent novel transcribed regions^2^,^3^,^4^, where transcripts longer than 200 nucleotides in length are localized. Much of this newly discovered major class of transcripts has very weak or no protein-coding potential and, thus, was defined as long intergenic non-coding RNAs (lincRNAs)^5^. Since the identification of the first two imprinted lincRNAs, H19 and Xist (X inactive specific transcript), in the early 1990s^6^,^7^, lincRNAs have emerged as an exciting new molecules with potential roles in a variety of cellular processes, including gene regulation^8^,^9^, X-chromosome inactivation^7^,^10^,^11^, reprogramming^12^, pluripotency maintenance^13^, embryonic development^14^ and paraspeckle formation^15^,^16^.

In recent decades, the main goal of pig breeding has been to improve the pig growth rate and muscularity^17^. Several studies have indicated that postnatal skeletal muscle growth is largely affected by prenatal skeletal muscle development^18^,^19^. Therefore, understanding the network dynamics of the muscle transcriptome during earlier fetal stages will be of great importance in unraveling the complex mechanism underlying muscle development. The vast majority of transcripts behave as non-coding RNAs (ncRNAs)^20^,^21^,^22^, which include a large portion of lincRNAs^23^,^24^. However, previous studies on porcine fetal skeletal muscle have primarily focused on protein-coding genes^25^,^26^,^27^,^28^ and miRNAs^29^,^30^,^31^,^32^ instead of lincRNAs; consequently, transcriptional information for skeletal muscle growth in swine is incomplete. Of note, previous investigations found that polycomb repressive complex 2 (PRC2) plays a key role in regulating myogenesis^33^ and that approximately 20% of lincRNAs are bound by PRC2^34^. These findings indicate that lincRNAs are most likely involved in the development of skeletal muscle.

Thousands of lincRNAs have been identified in humans and mice^5^,^35^, some of which demonstrate strong evolutionary signals of inter-species conservation^5^. However, the assembly of porcine lincRNA sequences using conserved lincRNAs as seeds for the collection of expressed sequence tags (ESTs) is still laborious and ineffective^36^. In particular, lincRNA transcripts obtained from different sources make it difficult to study their roles in skeletal muscle development. More recently, RNA-seq technology^37^ and computational methods developed for transcriptome reconstruction^35^,^38^ have facilitated comprehensive gene annotation and functional characterization of lincRNAs. These approaches have been successfully applied to identify and characterize lincRNAs in a given tissue or cell line^39^,^40^,^41^.

Muscle development in pig fetuses involves two major waves of fiber generation: primary fiber formation at 35–60 days post coitus (dpc) and secondary fiber formation at 54–90 dpc^42^. However, some studies have shown that the development of muscle fibers in the fetal pig is mostly complete by 70–75 dpc^43^,^44^, which is consistent with additional recent findings that porcine myogenesis is almost complete before 77 dpc^28^. Moreover, it was shown that the stage ranging from 50 to 75 dpc is critical for the formation of various muscle phenotypes^28^. Thus, the 50–75 dpc period is a critical stage of fetal skeletal muscle development.

In this study, we report the systematic identification and characterization of lincRNAs in porcine fetal skeletal muscle from paired-end RNA-seq data that were obtained from a pool of samples at 50, 55, 60, 65 and 75 dpc. We further characterized the basic features of a conserved mouse lincRNA, including its subcellular localization and its association with chromatin-modifying complexes. Our study paves the way for further studies exploring the functional roles of lincRNA during porcine skeletal muscle development.

## Results

### Read mapping and transcript assembly

A total of 55.02 million 90-bp pair-end reads were obtained after filtering out low-quality reads and removing the adaptor sequences. Approximately 73.1% of the total clean reads were mapped to the *Sus scrofa* genome assembly 10.2, and 54,550 assembled transcripts were produced.

### Genomic information of porcine lincRNAs

**Figure 1:**
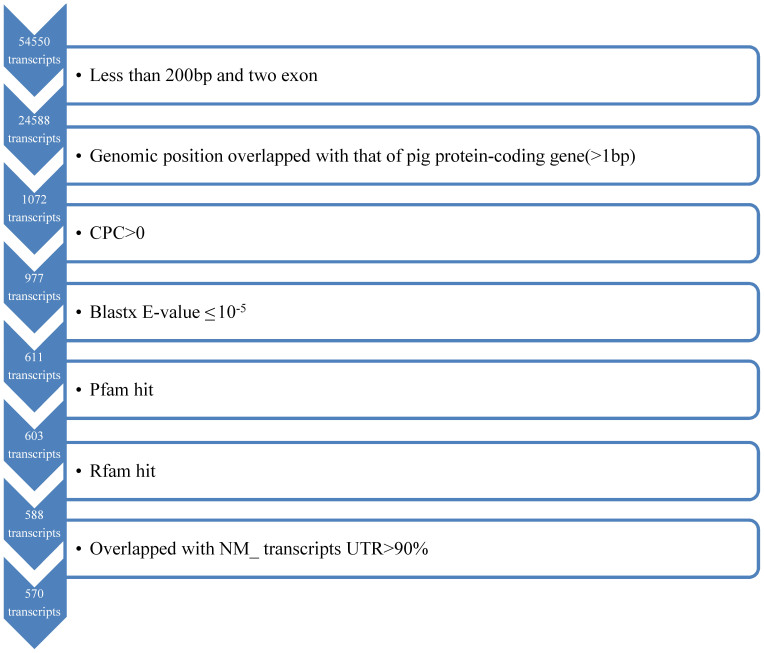
Overview of the stringent filtering pipeline used to identify the resulting 570 lincRNAs. At each step, the vertical arrow denotes the transcripts that passed the filter, and the box denotes those that were removed.

We developed a highly stringent filtering pipeline (Figure 1) to identify porcine lincRNAs using an integrated experimental and computational approach. In total, our pipeline yielded 570 lincRNA transcripts, corresponding to 476 lincRNA genes. Some lincRNA genes were alternatively spliced, containing 1.2 isoforms per lincRNA locus on average. We found that 45.4% of the total lincRNA was transcribed near (<10 kb) known protein-coding genes. The average size of porcine lincRNA was approximately 1,043 bp, with a range of 200 to 834 nucleotides that span 2.5 exons on average, which is similar to that of human lincRNA^39^. The average exon length of lincRNA was 417 bp, and lincRNAs that contained two exons accounted for 65.7% of the total lincRNAs. We also found that our lincRNA genes contained canonical splice sites (GT-AG); these lincRNAs were distributed in all chromosomes except the Y chromosome.

### Comparison between lincRNAs and protein-coding genes

**Figure 2:**
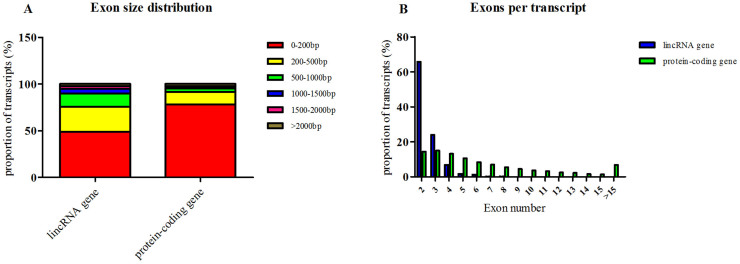
Comparison of features of porcine lincRNAs and protein-coding genes.

We also obtained 14,836 protein-coding transcripts that corresponded to 12,372 genes (an average of 1.2 isoforms per protein gene). The average length of these transcripts was 1,790 bp with an average of 6.8 exons, which was larger than the size of the lincRNA genes. However, the average exon length of the protein-coding genes was 261 bp, which was less than that of the lincRNA genes. Furthermore, the exon size distribution of protein genes was mostly within 200 bp (Figure 2A). We found that protein transcripts that contained only two exons accounted for 14.4% of the total protein-coding genes, which was far less than what was observed for the lincRNA genes (Figure 2B).

### Conservation of porcine lincRNAs

**Figure 3:**
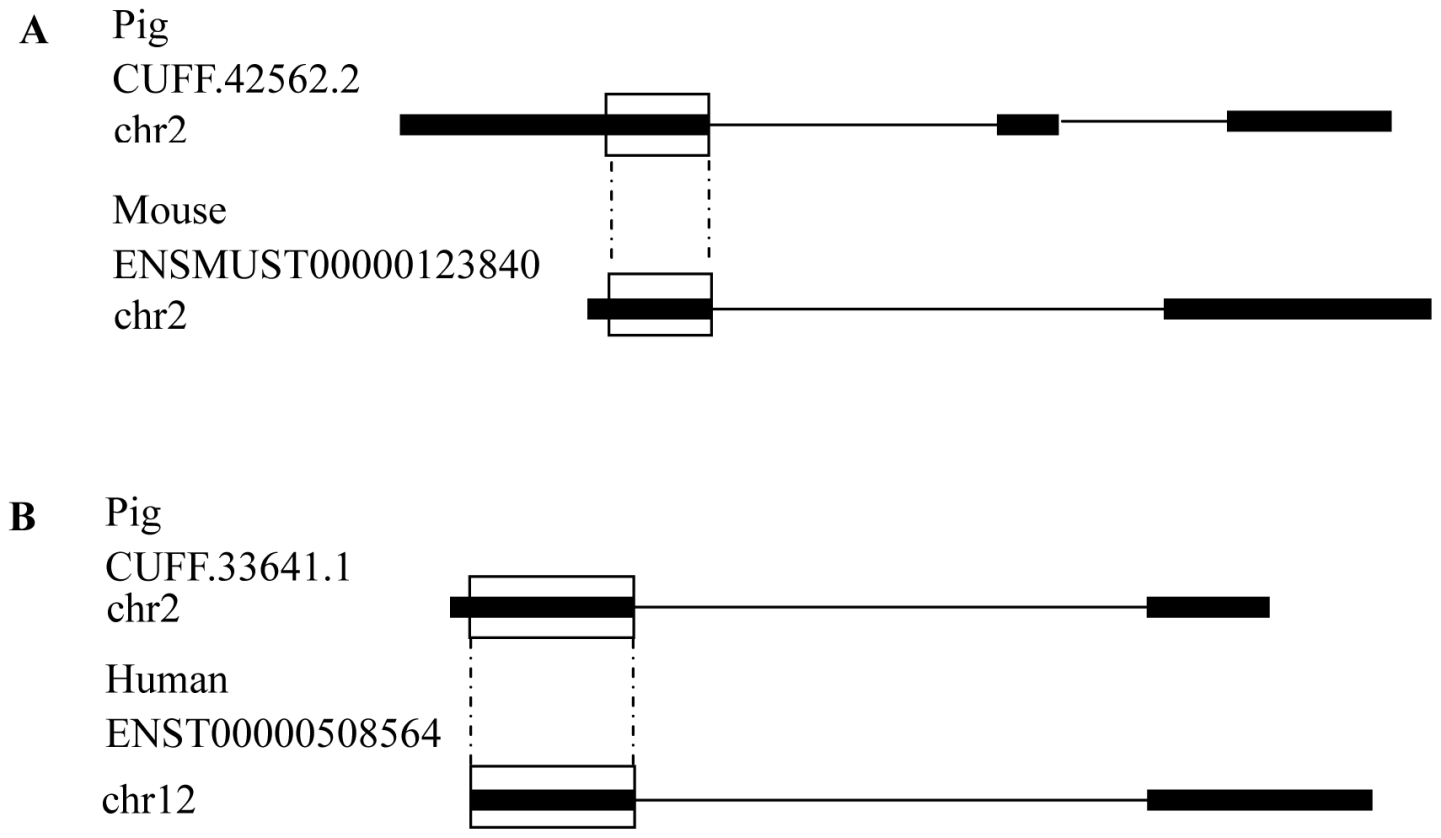
Representative images of two pig lincRNAs with conserved segments for mouse (A) and human (B). Thick lines indicate an exon, and thin lines indicate an intron of the lincRNA. Boxes indicate the conserved region between the two lincRNAs.

To examine the sequence conservation of lincRNAs between pig and other mammals, we compared pig lincRNAs with those of mouse and human lincRNAs (Ensembl Genes 70) using BLASTN version 2.2.26+. The Ensembl database contains 11,325 human lincRNAs and 3,148 mouse lincRNAs. Only 28 (5%) and six (1.05%) pig lincRNAs overlapped with human and mouse lincRNAs (E-value ≤10^−5^), respectively. Additionally, four of the lincRNAs corresponded to two lincRNA genes overlapping between human and mouse. The lincRNAs conserved between pig, human and mouse spanned a modest portion of the transcript ranging from 28 to 2,282 nt (396 nt on average) and 35 to 1,930 nt (612 nt on average), respectively. We further found that six and three pig lincRNAs had sequence homology with human and mouse lincRNAs, respectively, restricted to the regions located in a single exon in which the conserved regions started from or ended at the intron-exon boundary (Figure 3). In these nine cases, both the pig lincRNA and the mammalian ortholog were also spliced, indicating that the relative position of the exon within the conserved region was conserved.

In addition, we found that 364 and 137 porcine lincRNA loci could be synthetically mapped to the human and mouse genome using liftover with a value of 0.5 for the “Minimum ratio of bases that must remap”. Moreover, we found that 91 porcine lincRNA loci overlapped (>1 bp) with the human lincRNA loci, which also contained 19 lincRNAs of the above 28 lincRNAs. In addition, 20 porcine lincRNA loci overlapped (>1 bp) with the mouse lincRNA loci, which contained all of the above six lincRNAs.

### Repetitive elements of porcine lincRNA

**Figure 4:**
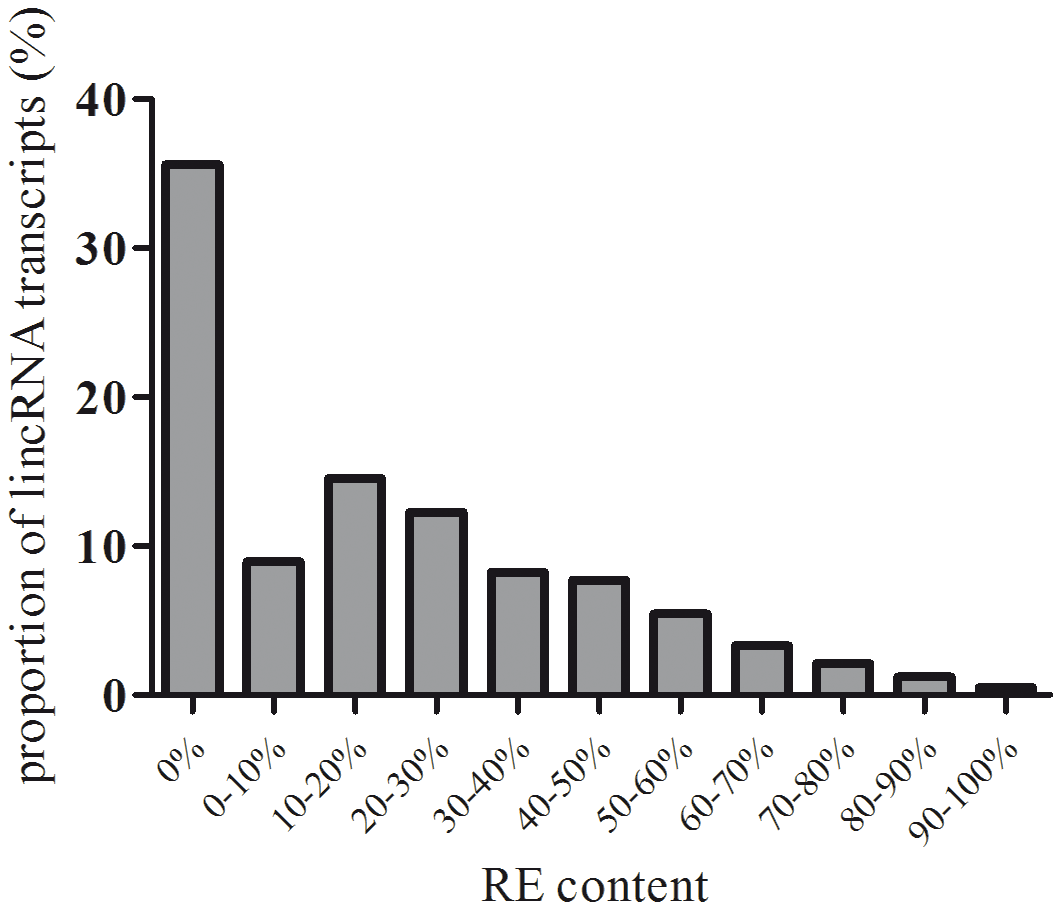
Percentage of pig lincRNA transcripts masked by RE (from 0 to 100%).

We found that 367 (65%) pig lincRNA transcripts harbored at least a partial repetitive element (RE) and that 12.64% of the lincRNA transcripts are composed of ≥50% RE-derived sequences (Figure 4). In general, the number of lincRNAs decreased with the increasing RE content ratio (Figure 4). The average size of the RE-derived fragments in the lincRNAs was 355 bp, whereas the average length of the 367 lincRNAs was 1,263 bp. Thus, on average, 28% of the lincRNA length is composed of REs. Short interspersed repetitive sequences (SINEs) and long interspersed repetitive sequences (LINEs) accounted for 57.64% of the total REs, and the long terminal repeat (LTR) occupied 15.52% of the total RE. In addition to the above major REs, 19.25% of REs were identified as simple repeat and low complexity sequences.

### Nearest neighbor analysis of lincRNA genes

We found that 259 lincRNA loci were transcribed near (<10 kb) their protein-coding neighbor, and a total of 378 protein-coding neighbors were collected. Of these neighbors, 361 were assigned to 26 GO terms involved in the biological process (Table 1, *P* < 0.05). The 26 GO terms mainly referred to the development process, transcriptional regulation and the biosynthetic process. We further found that all of the GO terms contained a small number of genes, except those related to transcription regulation and the biosynthetic process.

### Expression of lincRNAs at different developmental stages

**Figure 5:**
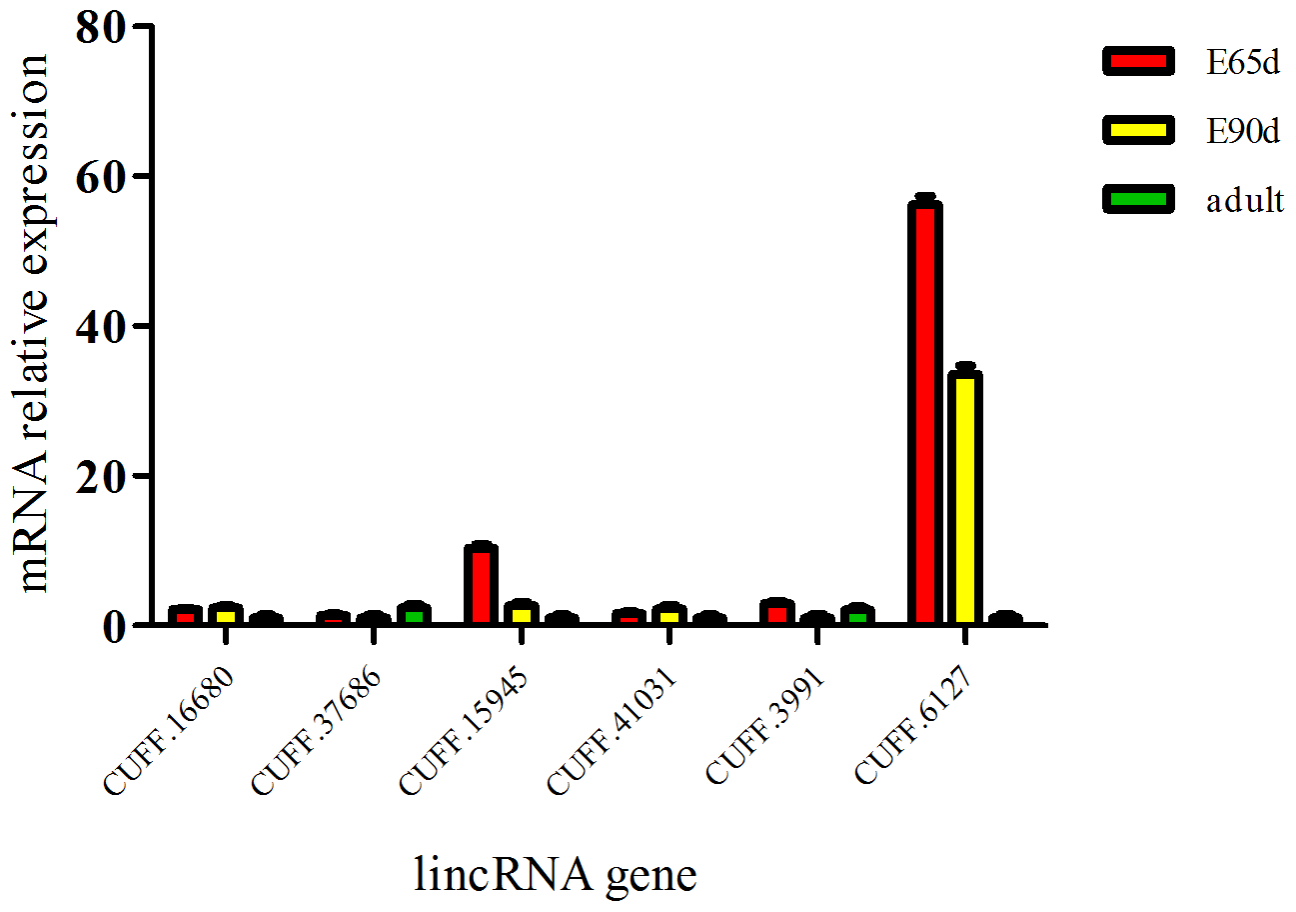
Developmental expression pattern of lincRNAs during muscle development (here and below, the values represent the means ± s.e.m., n = 6).

We randomly selected 10 lincRNAs and examined their expression pattern at three important development stages. The results confirmed the expression of seven lincRNAs, of which six were detected at all time points (Figure 5) and showed differential expression between the fetal and adult periods. We further found that CUFF.15945 and CUFF.6127 were both higher in the 65 dpc period and were considerably decreased during muscle development; they had a distinctive expression pattern compared with the other four lincRNAs.

### Characterization of ENSMUSG00000090086

**Figure 6:**
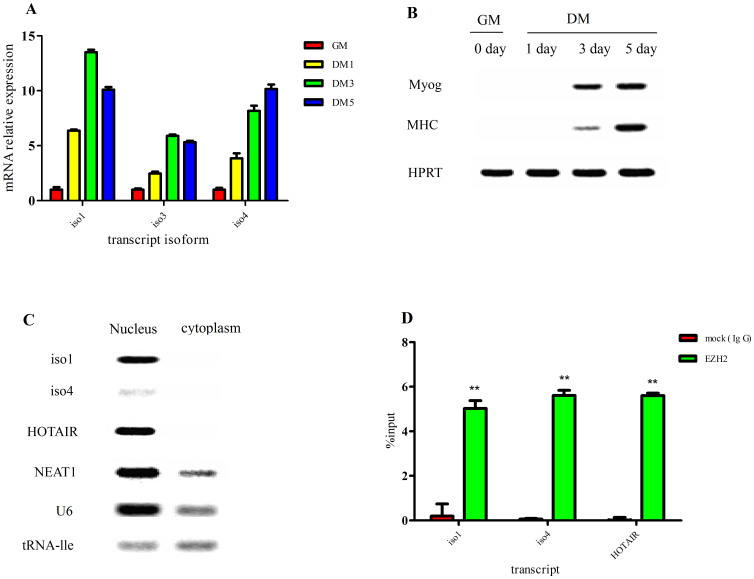
Features of the ENSMUSG00000090086 gene. (A) Q-PCR analysis of ENSMUSG00000090086 gene expression during C2C12 cell culture in growth medium (GM) or differentiation medium (DM) for 1, 3 and 5 days (here and below, the values represent the means ± s.e.m., n = 6). (B) RT-PCR analysis of Myog and MHC expression to monitor the differentiation status at indicated times. HPRT was used as an endogenous control. (C) RT-PCR analysis of the relative expression of iso1 and iso4 as well as other control genes in the nuclear and cytoplasmic cell fractions. (D) The RIP result of iso1, iso4 and HOTAIR with the EZH2 antibody. RIP enrichment was measured by q–PCR, and the values were normalized to background levels and input samples (Percent Input Method). The interaction of HOTAIR with EZH2 is a known interaction that served as a positive control(**indicates *P* < 0.01, here and below, the values represent the means ± s.e.m., n = 3).

We choose a mouse lincRNA gene locus (named ENSMUSG00000090086) that was conserved between pig and human to analyze its features during C2C12 cell differentiation. The ENSMUSG00000090086 gene contained seven transcript isoforms, and we found that iso3 and, in particular, iso1 and iso4 were considerably up-regulated during C2C12 cell differentiation; whereas iso2, iso5, iso6 and iso7 were not detected (Figure 6A). We separated the differentiated C2C12 cells into nuclear and cytoplasmic fractions and found that iso1 and iso4 were both expressed mainly in the nucleus, although iso4 showed weak expression a weak expression (Figure 6C, Supplementary Information). Further, in RNA immunoprecipitation (RIP) experiments, we confirmed that iso1 and iso4 were both significantly (*P* < 0.01) enriched with EZH2 antibody compared to the IgG nonspecific antibody (Figure 6D).

## Discussion

The identification and characterization of porcine lincRNA, particularly in fetal skeletal muscle development, has been very limited compared with that of lincRNAs in humans^39^,^45^ and other model organisms, such as zebrafish^14^,^46^ and mouse^5^,^35^. To the best of our knowledge, this is the first report of the systematic identification and characterization of a reference catalog of 570 porcine lincRNAs by integrating RNA-seq data from fetal muscle tissues. We annotated the basic features of the pig lincRNAs, including the transcript structure, sequence conservation, transposable elements, nearest neighbor analysis and developmental expression. Below, we discuss these findings in more detail and relate our findings to results that have emerged from humans and other model organisms.

Noncoding and protein-coding genes were distinguished by their coding potential capability. It has been reported that CPC can discriminate coding from noncoding transcripts with high accuracy^47^. Additionally, some reports have shown that the combination of a strict BlastX and Pfam (PfamA and PfamB) search could better reduce false negative and false positive results^39^,^46^. Therefore, we performed this combination of steps to ensure that our resultant lincRNAs were of high quality.

We noted that about 73.1% of the total clean reads were mapped to *Sus scrofa* genome assembly v.10.2, which showed a low efficiency of mapping. Here we only focus on the number of reads that could be mapped to all the 20 chromosomes (SSC1-18, X and Y), and, thus, we observed a low efficiency of mapping. Nevertheless, when the reads were mapped to the 20 chromosomes and unplaced scaffolds, the mapping rate was increased to 80.4%, which was in accordance with previous reports^48^.

LincRNA genes are typically shorter (~1 kb) and have fewer exons (~2–3) than protein-coding genes^39^,^46^ and, thus, have relatively simple compositions. Our putative porcine lincRNAs also display these properties, indicating that the lincRNAs identified here were reliable. However, an earlier study showed that porcine lincRNAs identified in the testis were 456 bp in size on average^40^, which is approximately half the size of the lincRNAs in human^39^ and swine characterized here. This inconsistency is possibly due to fewer reads and mapping problems. Our results showed that the porcine lincRNAs had 1.2 isoforms per locus, which was lower than that of human lincRNAs^39^. This difference does not seem to be attributable to the fewer reads, as zebrafish lincRNAs had more sequence reads compared to human lincRNAs, although they exhibited less efficient alternative splicing^39^,^46^. We also found that our lincRNAs had canonical splice sites (GT/AG), which supported the fact that the lincRNAs were similar to protein-coding genes in some properties, such as chromatin modification and splicing signals^39^,^49^.

We found that almost half of the porcine lincRNA was transcribed near (<10 kb) protein-coding genes, which is consistent with the finding that lincRNA genes were preferentially found within 10 kb of protein-coding genes^2^,^49^. However, some studies showed different results^50^, which may be attributed to the diverse sources of lincRNA. It was demonstrated that lincRNAs were transcribed in close proximity to protein-coding genes and was possibly coordinated with transcriptional regulation of neighboring coding genes^51^. This was partially supported by evidence that mammalian lincRNAs (<10 kb) are more likely to be located near genes that mediate transcriptional regulation^14^,^39^, which was also observed in the GO analysis in the nearest neighbors of porcine lincRNA genes.

Transposable elements comprise a substantial fraction of the vertebrate genome^1^ and have been shown to be a major source of vertebrate lncRNAs^52^. In this study, most of the porcine lincRNAs were also composed of partial TE-derived sequences, which is consistent with the above conclusion. Moreover, we found that SINEs and LINEs account for half of the TE family in the porcine lincRNAs, which is also consistent with the results observed in the mammalian lncRNAs^52^.

Most of the lincRNAs had a less conserved sequence with other mammalian lincRNAs, and some of them had more positional conservation than sequence conservation across vertebrates^14^. Our results showed that few pig lincRNAs overlapped with the human and mouse lincRNAs at the sequence level, but more pig lincRNAs were synthetically mapped to the mammalian genome, which is consistent with the above conclusions. However, a recent report showed that nearly 40% of pig lincRNAs had detectable sequence homology with human and mouse lincRNAs by BLASTN. We found that our pig lincRNAs were only obtained from the skeletal muscle, while the 6,621 pig lincRNAs in Zhou et al. (2014) were obtained from several tissues^53^. LincRNAs exhibited tissue-specific expression patterns more so than protein-coding genes, which may lead to identify different number and structure of lincRNAs across multiple tissues. Furthermore, the mouse lincRNAs from the NONCODE database (v4)^54^ and human lincRNAs from the Gencode database (v19)^55^ database was also different from the mouse and human lincRNAs database (Ensemble Genes 70) analysed in this study. Thus, we think the difference in the number and source of pig lincRNAs and the databases of mouse and human lincRNAs may have led to our poor results about lincRNAs sequence similarity between species.

Conserved lincRNAs among mammals are generally thought to have important roles^14^. We found that the conserved ENSMUSG00000090086 gene was differentially expressed during C2C12 cell differentiation and was associated with PRC2. It was shown that the binding of differentially expressed lincRNAs to PRC2 could indicate a possible role of lincRNAs^8^. However, a recent study stated that the binding to PRC2 does not necessarily imply functionality for lincRNA^56^. The role of the ENSMUSG00000090086 gene needs to be further investigated.

In conclusion, we have provided a resource of porcine lincRNA which will enable further studies of the function of these genes in the process of skeletal muscle development.

## Methods

The methods were performed in accordance with the guidelines of the Good Experimental Practices adopted by the Institute of Animal Science.

All experimental protocols were approved by the Institute of Animal Science of the Chinese Academy of Agricultural Sciences.

### Animal and tissue preparation

All longissimus dorsi muscle samples, which were maintained in liquid nitrogen, were derived from our laboratory. Two Tongchen pig fetuses (one male and one female) at 50, 55, 60, 65 and 75 dpc were included in this study.

### RNA extraction, library preparation and Solexa sequencing

Total RNA was isolated with TRIzol reagent (Invitrogen, Carlsbad, CA, USA), treated with DNase I (Qiagen, Beijing, China), and purified using an RNeasy MinElute Cleanup column (Qiagen). The total RNA integrity was assessed using Agilent 2100 Bioanalyzer (Agilent Technologies, Palo Alto, CA, USA), and only the samples with RNA Integrity Number (RIN) scores >8 were used for sequencing. Equal amounts of total RNA from the samples at the different stages (*i.e.*, 50, 55, 60, 65 and 75 dpc) were pooled into one sample.

PolyA^+^ RNA was purified using Magnetic Oligo (dT) Beads from 20 μg of the total RNA and was further fragmented before cDNA synthesis. First-strand cDNA was synthesized using Random Primer p(dN)_6_ and Superscript III (Invitrogen, Carlsbad, CA, USA), and the synthesis of double-stranded cDNA was performed using 10× second strand buffer, RNaseH, and DNA Polymerase I. Following the second-strand cDNA synthesis and adaptor ligation, 240–310 bp cDNA fragments were isolated. The cDNA libraries were then prepared following the manufacturer's instructions (Illumina, San Diego, CA, USA). The purified cDNA libraries were sequenced using a paired-end sequencing strategy on the Illumina HiSeq 2000 after quantification by the Agilent 2100 Bioanalyzer.

### Transcriptome assembly

The raw reads were cleaned by filtering the adapter using cutadapt v1.1^57^ and low-quality reads using Prinseq v0.17.3^58^. The clean reads were then mapped to the pig reference genome (Sscrofa10.2) using the TopHat version 1.3.2 software^59^. Transcriptomes were assembled with Cufflinks version 1.3.0^38^ supported through Galaxy (https://usegalaxy.org/u/jeremy/w/sort-sam-file-for-cufflinks).

### Pipeline for the identification of multiple-exon lincRNA

We identified multiple-exon lincRNAs following the steps listed in the pipeline (Figure 1). The steps are detailed as follows:

(1) Size selection: single-exon transcripts and the transcripts less than 200 bp were removed; (2) the remaining transcripts were removed if they had genomic positions that overlapped (>1 bp) with those of pig protein-coding genes obtained from NCBI RefSeq mRNAs (release 54, July 2012) with the accession prefixes NM_ and XM_ (hypothetical protein genes were not included) and Ensembl protein-coding genes (Ensembl release 68, July 2012); (3) the Coding Potential Calculator (CPC) tool^47^ was used to assess the coding potential of transcripts in both strands, and the remaining transcripts were removed if they had a CPC value >0 in either strand; (4) any remaining transcripts with similarity to known proteins against the Swiss-Prot database with an E-value ≤10^−5^ were removed using the NCBI BLAST version 2.2.26; (5) the remaining transcripts that contained a known protein-coding domain were removed. To accomplish this, we translated each transcript sequence in all six possible frames and used HMMER to exclude the transcripts whose corresponding translated protein sequences had a significant hit in the Pfam (PfamA and PfamB) database release 26.0^60^; (6) the remaining transcripts that belonged to known classes of small RNAs (snRNA, snoRNAs, tRNAs, miRNA, etc.) were removed using Rfam^61^ (release 10.0); and (7) to filter the transcripts that were located in the UTR regions of the protein-gene due to incomplete assemblies, the remaining sequences were aligned against the NCBI RNA reference sequences (RefSeqs) only with the identifiers beginning with “NM_” prefixes via BLASTN. The sequences with more than 90% of their lengths overlapping in the UTR regions of the RNA RefSeqs were discarded.

### Analysis of protein-coding transcript, TEs and GO

The assembled transcripts that had at least two exons were collected and were considered to be protein-coding transcripts when they had a sequence similarity of ≥96% and overlapped with ≥90% of the porcine protein genes from NCBI RefSeq mRNAs (hypothetical protein genes were not included; release 54, July 2012) and Ensemble (release 68, July 2012) using BLASTN version 2.2.26+ (ftp://ftp.ncbi.nlm.nih.gov/blast/executables/blast+/LATEST/).

We ran RepeatMasker program version open-4.0.3 (http://www.repeatmasker.org/) with options “cross_match” as the search engine and “pig” as the DNA source to identify transposable and repetitive DNA elements in the pig lincRNA sequences.

For each lincRNA locus, the nearest upstream and downstream (within <10 kb) protein-coding neighbors (without overlap) were identified. The neighbor gene names were used as the gene list input into DAVID (http://david.abcc.ncifcrf.gov/) for GO analysis^62^. We selected the “GOTERM_BP_FAT” and set the value of EASE to 0.05 for the GO term enrichment analysis.

### Reverse transcription polymerase chain reaction (RT-PCR) and real-time PCR

For RT-PCR, the total RNA was converted into cDNA using a Revert Aid First Strand cDNA Synthesis Kit (Thermo Fisher Scientific, Boston, MA, USA) with oligo dT and random hexamer primers included in the kit. The PCR reactions were performed as follows: initial denaturation at 95°C for 3 min, followed by 30 cycles of denaturation at 95°C for 15 s, annealing at 60°C for 30 s, and elongation at 72°C for 20 s. The real-time PCR was performed according to the SYBR Premix Ex Taq™ instructions (Takara, Shiga, Japan). The reaction volume contained 10 μl of 2× SYBR® Premix Ex Taq™, 0.4 μl of 50× ROX Reference Dye II, 0.5 μl of 10 μM forward and reverse primers, 2 μl of template cDNA and dH_2_O up to a final volume of 20 μl. The reactions were performed on an ABI 7500 instrument (Applied Biosystems, Carlsbad, CA, USA) as follows: 2 min at 95°C, followed by 35 cycles of 5 s at 95°C and 34 s at 60°C. All data were analyzed by the 2−ΔΔCT method using 7500 System (SDS) Software version 1.4.0. LincRNA primers (Table 2) were designed over suitable exon-exon junctions using primer3 (http://bioinfo.ut.ee/primer3-0.4.0/primer3/input.htm).

### Cell culture and differentiation

The mouse C2C12 cell line was provided by the Cell Resource Center of Peking Union Medical College (CRC/PUMC, Beijing, China) and was cultured in Dulbecco's modified Eagle's medium (DMEM) - high glucose (Invitrogen, Carlsbad, CA, USA) with 10% (v/v) fetal bovine serum (Invitrogen, Carlsbad, CA, USA) at 37°C in a 5% CO_2_ humidified incubator. Upon the induction of differentiation, the culture medium was switched to DMEM plus 2% horse serum when cells reached approximately 80% confluence. The medium was changed every two days.

### Nuclear and cytoplasmic RNA fractionation

The cells cultured in differentiation medium were harvested in a T-25 flask, washed once with cold PBS, and centrifuged at 500 g for 3 min at 4°C. Cell pellets were resuspended by gentle pipetting in 200 μl of lysis buffer (10 mM Tris-HCl, pH = 8.0, 140 mM NaCl, 1.5 mM MgCl_2_, 10 mM EDTA, 0.5% IGEPAL® CA-630, and 40 U/ml RNase inhibitor) and incubated on ice for 5 min, followed by centrifugation at 500 g at 4°C for 3 min. The supernatant was transferred to a fresh 1.5 ml microcentrifuge tube, centrifuged at full speed (14,000 rpm) for 1 min, and lysed in 1 ml of TRIzol for cytoplasmic RNA isolation. The nuclear pellet was washed once with lysis buffer and was resuspended in 200 μl of lysis buffer to determine the nucleus viscosity; 1 ml of TRIzol was added for nuclear RNA isolation if necessary. An equal amount of nuclear and cytoplasmic RNA was reverse transcribed for further analysis.

### RNA-binding protein immunoprecipitation (RIP)

Cells cultured in differentiation medium were prepared in four T-75 flasks for the RNA-binding protein immunoprecipitation (RIP) assay using an EZ-Magna RIP Kit (Millipore, Billerica, MA, USA) and following the manufacturer's instructions. An anti-EZH2 polyclonal antibody (ab3748, 1:100; Abcam, Cambridge, UK) and negative control rabbit IgG antibody was used to investigate the interactions between lincRNAs and PRC2. The final isolated RNA was reverse transcribed using random primers according to the RevertAid™ First-Strand cDNA Synthesis Kit. Data were analyzed using the Percent Input Method (http://www.lifetechnologies.com/cn/zh/home/life-science/epigenetics-noncoding-rna-research/chromatin-remodeling/chromatin-immunoprecipitation-chip/chip-analysis.html).

### Statistical analysis

The results are reported as the mean ± standard deviation (SD). Statistical analysis was performed using a two-tailed unpaired Student's t-test. Differences were considered statistically significant at the p < 0.05 level and were considered very significant at p < 0.01. All experiments were performed three times.

## Supplementary Material

Supplementary Information Supplementary Information

## Figures and Tables

**Table 1:** GO analysis of the closely neighboring protein-coding genes of lincRNA

No.	Terms	GO Accession	No. of genes
1	skeletal muscle organ development	GO:0060538	7
2	skeletal muscle tissue development	GO:0007519	7
3	anterior/posterior pattern formation	GO:0009952	9
4	pattern specification process	GO:0007389	12
5	regionalization	GO:0003002	10
6	segmentation	GO:0035282	5
7	regulation of transcription from RNA polymerase II promoter	GO:0006357	23
8	tongue development	GO:0043586	3
9	segment specification	GO:0007379	3
10	striated muscle tissue development	GO:0014706	7
11	cell fate determination	GO:0001709	4
12	regulation of transcription	GO:0045449	61
13	muscle tissue development	GO:0060537	7
14	leukocyte activation	GO:0045321	10
15	regulation of membrane potential	GO:0042391	7
16	negative regulation of protein catabolic process	GO:0042177	3
17	positive regulation of transcription from RNA polymerase II	GO:0045944	13
18	blood vessel morphogenesis	GO:0048514	9
19	muscle organ development	GO:0007517	9
20	regulation of transcription, DNA-dependent	GO:0006355	43
21	positive regulation of cellular biosynthetic process	GO:0031328	20
22	positive regulation of nitrogen compound metabolic process	GO:0051173	19
23	autonomic nervous system development	GO:0048483	3
24	cranial nerve development	GO:0021545	3
25	negative regulation of cellular protein metabolic process	GO:0032269	8
26	positive regulation of biosynthetic process	GO:0009891	20

**Table 2:** LincRNA primers used in this study

Primers	Primer sequence		Amplicon size (bp)
	Forward (5′-3′)	Reverse (3′-5′)	
CUFF30734	CCCTCTTCATTTCACCAGGA	GCAGGCTGAGGACGAGAATA	192
CUFF.34686	ATATTCCTCCCGGGTTTCAC	CCACAGCCAGAATCATCCTT	218
CUFF.48491	GACTACCATCTTGGGGACCA	TGAAGAACCGGGGTTATCAG	210
CUFF.15945	CTAAGGCCCTCTGCAAACTG	TTTTGCTTCCAACTTTTCCA	239
CUFF.6127	CAATGCTGTGGCAACAAGAC	CAGACGAAAGCCAGAAGTCC	241
CUFF.41031	CCCAAGGTGGTGGTTTAGTG	CCCCGTTACTGTGGTACCTG	224
CUFF.3991	ATCCATCCAGCATCTTCTCG	GACACGTGCCAGGTACTGAG	227
CUFF.16680	ACACGTGTGCCAGTCAACAT	AGCCCATCAGTCCCTCTTCT	157
CUFF.1689	GTGCAGCCACTTCTTTCTCC	TTGCAGGTCCCATTCCATAC	228
CUFF.37686	AGGGCCCTCAGTTGTGATTT	GTGGGTGAAGGATTTCCCTA	174
Pig-TBP	GCGATTTGCTGCTGTAATCA	CCCCACCATGTTCTGAATCT	196
Iso1	GTATTTTCTGTGGCGGTTGG	GATGTCCAGAGGAAGCAAGG	282
Iso3	GGAATTGCCCTAACAGAACG	CTACCCGGCCTAGAGTGTTG	210
Iso4	ACTCCACCATCCAGTTCAGG	TTCGCTGAGGGCTGTTTAGT	216
Myog	ACTCCCTTACGTCCATCGTG	CAGGGCTGTTTTCTGGACAT	195
MHC	CGTCAAGGGTCTTCGTAAGC	ATTGTTCCTCAGCCTCCTCA	158
U6	CGCTTCGGCAGCACATATAC	TTCACGAATTTGCGTGTCAT	87
tRNA-lle	AGTGGCGCAATCGGTTAG	AGGCTCGAACTCACAACCTC	78
NEAT1	TTTGAGATGCAGTTGCTTGG	CTCCCCAGCTTCACTTCTTG	205
HOTAIR	GGGCTGCAGAATTCACTCTC	GACTTCCTTCCTTCCGCTCT	207
HPRT	GCCCCAAAATGGTTAAGGTT	TTGCGCTCATCTTAGGCTTT	208
