# Supplementary material for: Systematic identification and characterization of long intergenic non-coding RNAs in fetal porcine skeletal muscle development
Source: Sci Rep. 2015 Mar 10;5:8957. doi: 10.1038/srep08957 (PMC4354164; doi:10.1038/srep08957)

Title:

Systematic identification and characterization of long intergenic non-coding RNAs in fetal porcine skeletal muscle development

Author list:

Weimin Zhao^1*^, Yulian Mu^1*^, Lei Ma^1^, Chen Wang^1^, Zhonglin Tang^1^, Shulin Yang^1^, Rong Zhou^1^, Xiaoju Hu^2, 3^,Menghua Li^2^ & Kui Li^1^

full-length gels of Myog and MHC


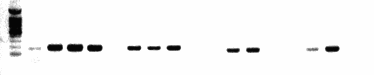


full-length gels of HPRT



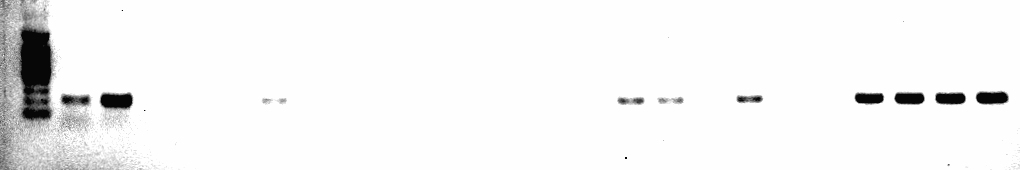


full-length gels of HOTAIR and NEAT1

N: nuclear C: cytoplasm


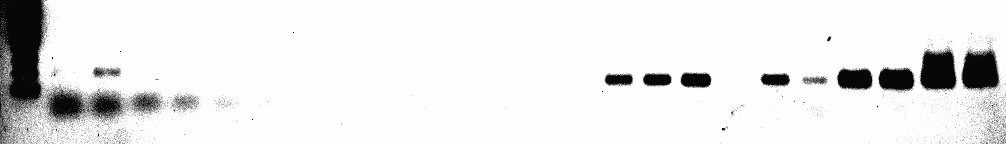


full-length gels of iso1, iso4, U6 and tRNA-lle

N: nuclear C: cytoplasm


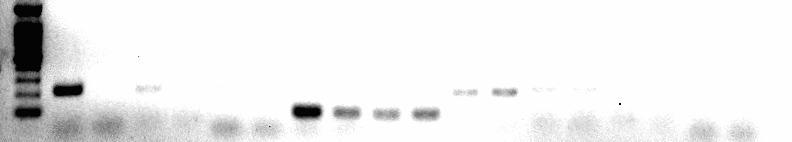


Supplement: Supplementary Information [file srep08957-s1.docx]
